# Generalized adjacency and the conservation of gene clusters in genetic networks defined by synthetic lethals

**DOI:** 10.1186/1471-2105-13-S9-S8

**Published:** 2012-06-11

**Authors:** Zhenyu Yang, David Sankoff

**Affiliations:** 1 Department of Mathematics and Statistics, University of Ottawa, 585 King Edward Avenue, Ottawa, Canada K1N 6N5

## Abstract

**Background:**

Given genetic networks derived from two genomes, it may be difficult to decide if their local structures are similar enough in both genomes to infer some ancestral configuration or some conserved functional relationships. Current methods all depend on searching for identical substructures.

**Methods:**

We explore a generalized vertex proximity criterion, and present analytic and probability results for the comparison of random lattice networks.

**Results:**

We apply this criterion to the comparison of the genetic networks of two evolutionarily divergent yeasts, *Saccharomyces cerevisiae *and *Schizosaccharomyces pombe*, derived using the Synthetic Genetic Array screen. We show that the overlapping parts of the networks of the two yeasts share a common structure beyond the shared edges. This may be due to their conservation of redundant pathways containing many synthetic lethal pairs of genes.

**Conclusions:**

Detecting the shared generalized adjacency clusters in the genetic networks of the two yeasts show that this analytical construct can be a useful tool in probing conserved network structure across divergent genomes.

## Introduction

As two related organisms diverge through evolutionary time, functional relationships among genes may alter. Some relationships may weaken, others strengthen, some may disappear while new ones appear. New genes or variants of genes may take on specific functions, while other genes may be inactivated or lost. And these changes proceed independently in the two evolving species. Even if most changes are local, affecting one or two relationships and two or three genes, after a long enough period of time the inventory of relationships in each of the species may reflect relatively little of the original pattern in the common ancestor, and may be quite different from each other.

Given two graphs representing functional genetic networks of two organisms, then, it may be difficult to decide if the local structures are similar enough in both graphs to infer some ancestral configuration or some conserved functional relationships. Current methods all depend on searching for identical substructures [1]. We have recently explored the notion of *generalized adjacency *to compare chromosomal gene ordering in two or more genomes [2-4] as way of parametrizing the relative importance of conserved gene order versus total gene content within a cluster. However, this concept is not tied to the physical nature of chromosomes; it has a graph-theoretical definition based solely on the adjacency of pairs of genes as a consequence their linear order along the chromosome. As such it is applicable to more general graphs. In this paper we will use generalized adjacency to compare the genetic networks of two species, representing the functional interaction between their genes.

Our work falls in the tradition of situating *small world *networks between regular lattice structures, with their dense local connections throughout, and completely random graphs with their short characteristic path lengths. Small world networks tend to have both properties, as discussed by Goldberg and Roth [5]. In the next section we define generalized vertex adjacency in a graph, and generalized adjacency clusters. Since these definitions involve a parameter, we invoke our previous work on finding a "natural" value for this parameter, and discuss its application to networks. We then sketch some analytic results on the distribution of the number of generalized adjacencies in the comparison of two randomly labelled regular lattices, and propose a general result for the comparison of two arbitrary graphs on the same set of vertices.

**Figure 1:**
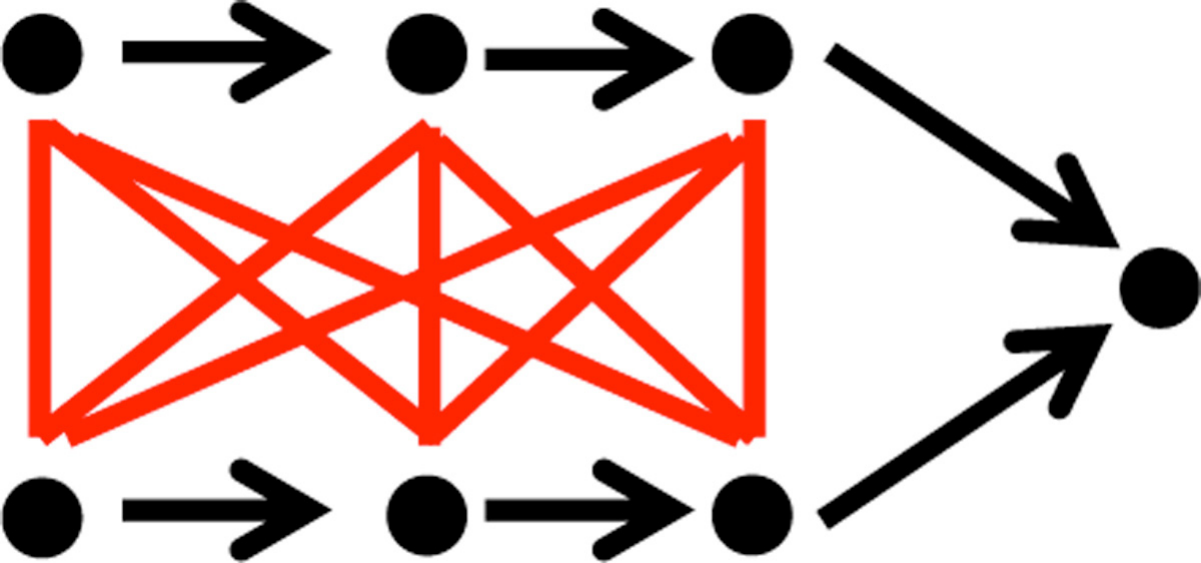
**Synthetic lethal**. Parallel pathways, indicated by arrows, converging at a single gene in a genetic network. Genes represented by dots. Synthetic lethal pairs of genes connected by red lines.

We apply our concepts to the comparison of genetic networks of *Saccharomyces cerevisiae *and *Schizosaccharomyces pombe*. The networks were obtained using Synthetic Genetic Array screens for "synthetic lethals" among virtually all pairs of genes whose individual inactivation is not lethal [6-8]. Typically, these pairs are organized in two parallel pathways that converge on a common endpoint, as illustrated in Figure 1. These pathways buffer each other so that the inactivation of one or more genes on a single one of the pathways will not affect survival, but inactivating at least one gene on *both *pathways is lethal.

We discover a pattern of local clustering in the edges common to both networks beyond what is defined by vertex adjacency alone. We suggest this is a consequence of the synthetic lethals methodology for building the networks.

## Methods

### Generalized vertex adjacency

Let *S *be a gene network with a gene set *V *= {1,..., *n*}. Two genes *g *and *h *are *i-adjacent*, and the pair (*g, h*) is an *i-adjacency*, in the gene network *S*, written in $g\overset{i}{\sim }h$ in *S*, if there are *i - *1 genes between them in *S *along a shortest path from one gene to the other. We define genes *g *and *h *to be (*i, j*)*-adjacent*, and the pair (*g, h*) is called an (*i, j*)*-adjacency*, in two gene networks *S *and *T*, if they are *i*-adjacent in either one of the gene networks and *j*-adjacent in the other. We say *g *is an *i-adjacent neighbor *of the gene *h *in a gene network *S*, if *g *and *h *are *i*-adjacent in *S*.

We denote $E_{M}^{\Theta }$ the set of all *i *adjacencies in a network *M *, where 1 ≤ *i *≤ Θ For two networks *S *and *T *with the same vertex set *V *= {1,..., *n*}, we define a subset of *C *⊆ *V *to be a (*θ, ψ*) *generalized adjacency cluster*, or (*θ, ψ*) *cluster*, if all vertices in the subset *C *are also the whole vertices of a connected component of the graph $G_{ST}^{\theta \psi }=\left(V,\left(E_{S}^{\theta }\cap E_{T}^{\psi }\right)\cup \left(E_{S}^{\psi }\cap E_{T}^{\theta }\right)\right).$

**Figure 2:**
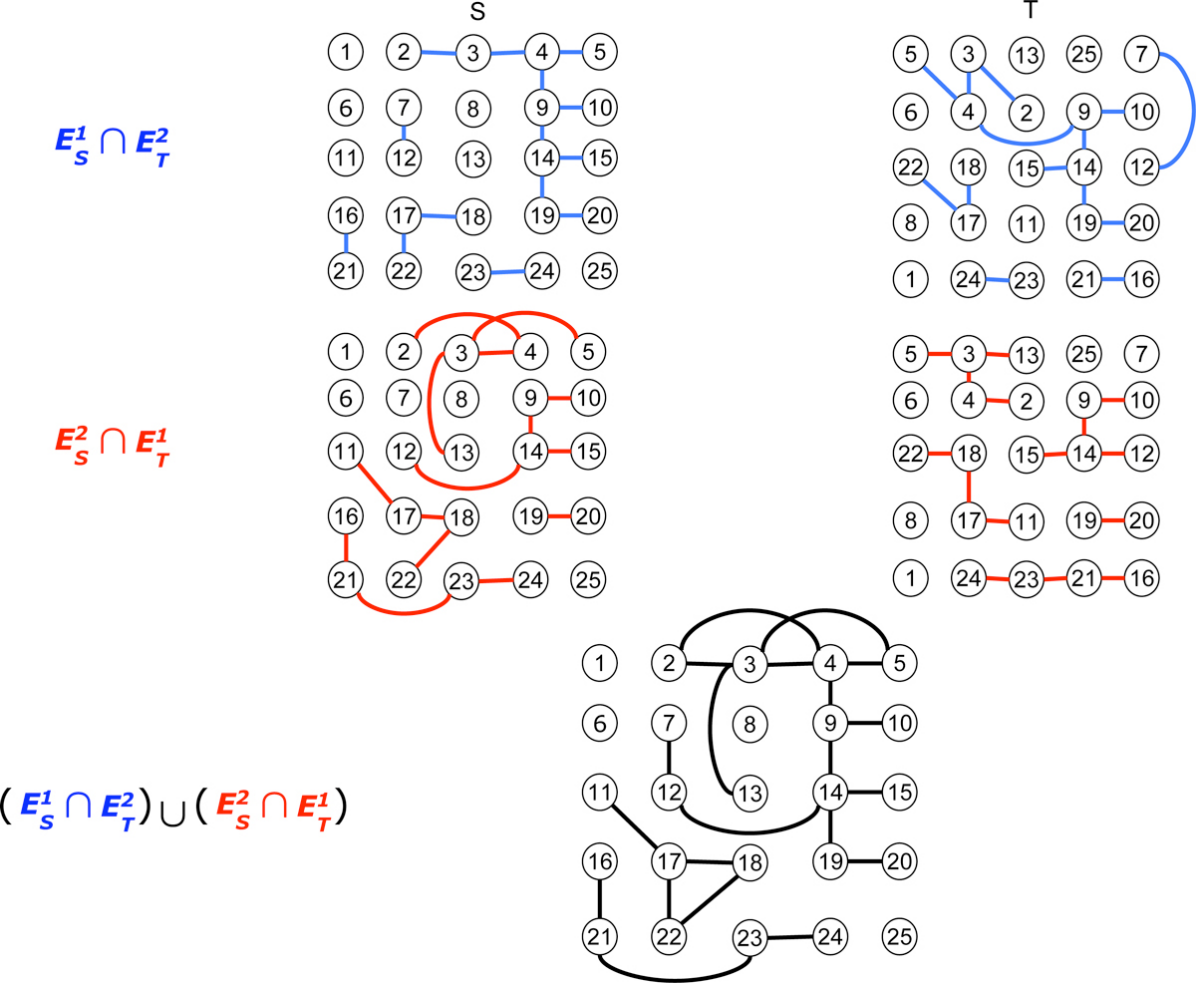
**Square grid**. Determination of (1, 2) clusters (or (2, 1) clusters) in square grid graphs and the clusters are {2, 3, 4, 5, 7, 9, 10, 12, 13, 14, 15, 19, 20}, {11, 17, 18, 22} and {16, 21, 23, 24}.

**Figure 3:**
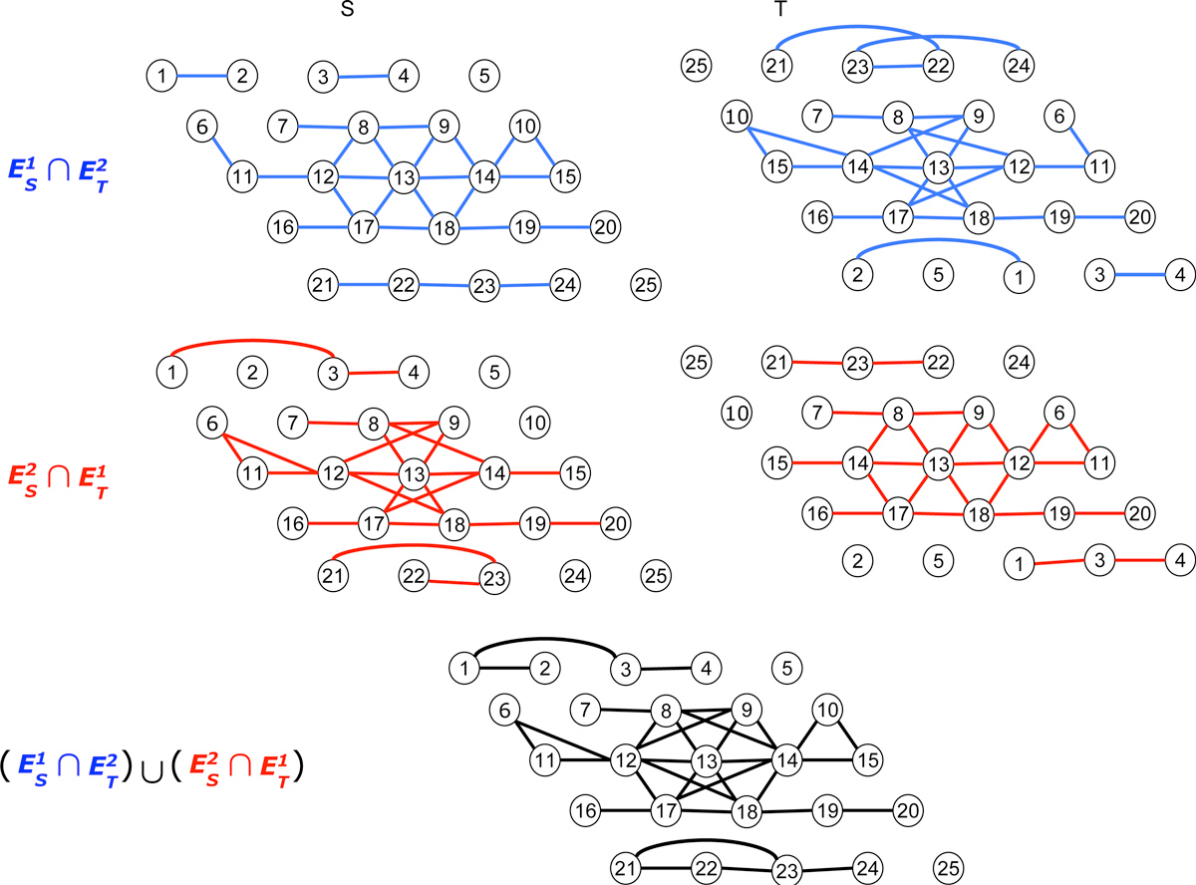
**Triangular grid**. Determination of (1, 2) clusters (or (2,1) clusters) in triangular grid graphs and the clusters are {1, 2, 3, 4}, {6, 7, 8, 9, 10, 11, 12, 13, 14, 15, 16, 17, 18, 19, 20} and {21, 22, 23, 24}.

**Figure 4:**
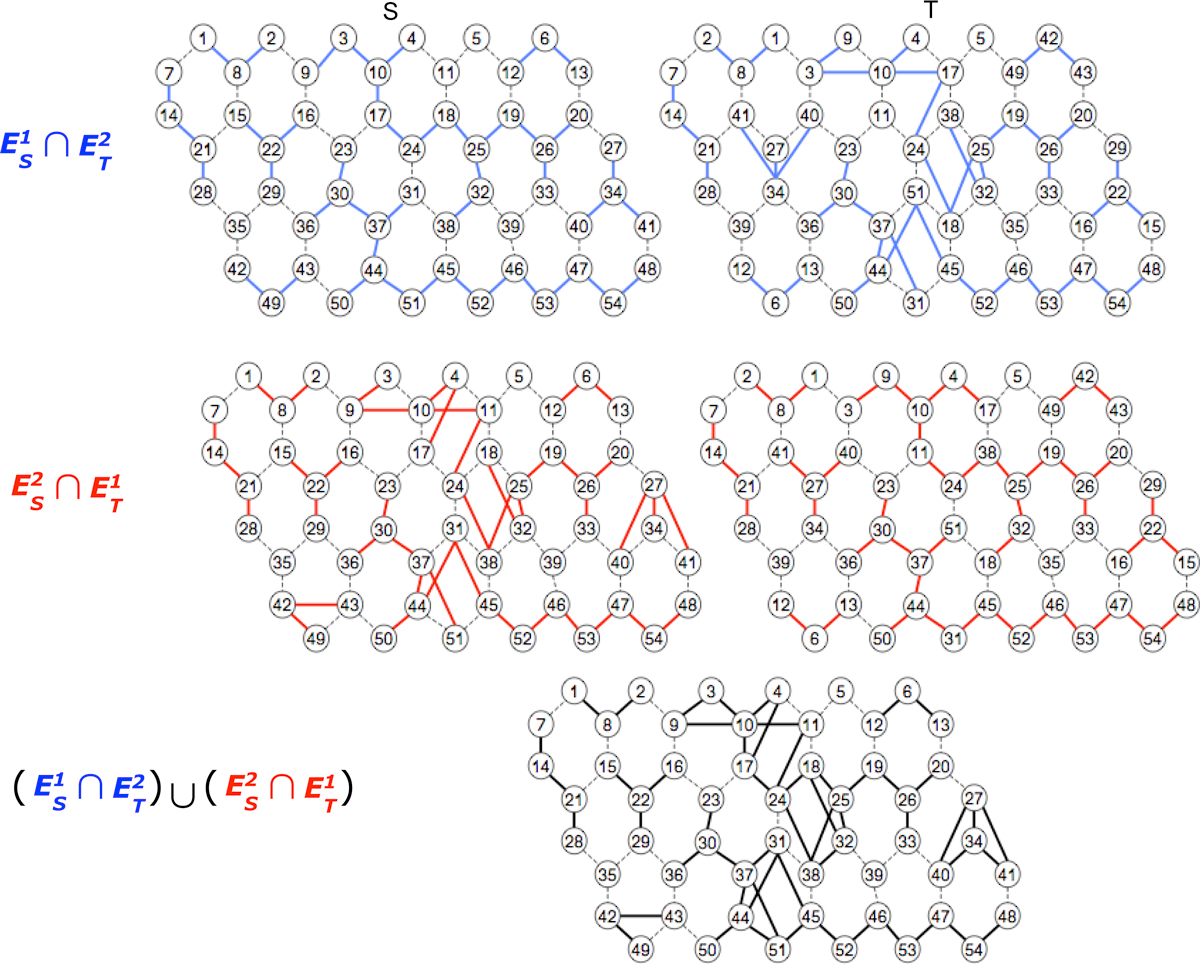
**Hexagonal grid**. Determination of (1, 2) clusters (or (2, 1) clusters) in hexagon grid graphs and the clusters are {1, 2, 8}, {6, 12, 13}, {27, 34, 40, 41}, {7, 14, 21, 28}, {15, 16, 22, 29}, {43, 44, 49}, {3, 4, 9, 10, 11, 17, 18, 19, 20, 24, 25, 26, 32, 33, 38} and {23, 30, 31, 36, 37, 44, 45, 46, 47, 48, 50, 51, 52, 53, 54}.

To obtain (*θ, ψ*) clusters of two gene networks, *S *and *T*, the new network $G_{ST}^{\theta \psi }$ need to be created first. The network $G_{ST}^{\theta \psi }$ can be constructed by connecting two genes of gene networks *S *and *T *if they are *i*-adjacent in *S *and *j*-adjacent in *T*, where max(*i, j*) ≤ max (*θ, ψ*) and min(*i, j*) ≤ min (*θ, ψ*). Figure 2 illustrates how the grid networks *S *and *T *determine the (1, 2) clusters {2,3,4,5,7,9,10,12,13,14,15,19,20},{11,17,18,22} and {16,21,23,24}. Figures 3 and 4 depict the same process for triangular graphs and hexagonal graphs, respectively.

### Weight function

The definition of generalized adjacency cluster in the previous section does not discriminate among pairs of (*i, j*)-adjacent genes as long as *i *and *j *are less than some cut-off values. However, it seems reasonable to think that (*i, j*) with smaller *i *and *j *should be weighted more heavily in defining clusters. To explore this, consider two networks *S *and *T *with the same vertices. Let *w_ij _*be the *weight *on two vertices that are (*i, j*)-adjacent, i.e., *i*-adjacent in one of the networks and *j*-adjacent in the other, such that

1. 0 ≤ *ω*_*ij *_= *ω_ji_, i, j *∈ {1, 2,..., *n*-1}

2. $\sum_{i=1}^{n-1}\sum_{j=1}^{n-1}\omega_{ij}=1$

3. *ω_i, j _*≥ *ω_k, l _*if

(a) max(*i, j*) *<*max(*k, l*) or

(b) max(*i, j*) = max(*k, l*) and min(*i, j*) *<*min(*k, l*)

This is a very general class of weights with reasonable monotonicity and total weight conditions. We define the *dissimilarity *between two gene networks *S *and *T *as

(1) $$d\left(S,T\right)=2P-\overset{l}{\underset{i=1}{\sum }}\left(n_{ii}\omega_{ii}+\overset{l}{\underset{j=1}{\sum }}n_{ij}\omega_{ij}\right).$$

where *P *is the number of pairs (*x, y*) that are (1, 1)-adjacent in two identical gene networks. *n_ij _*is the total number of pairs (*x, y*) that are *i*-adjacent in *S *and *j*-adjacent in *T*. *l *is the diameter of the network. We have argued elsewhere [4] that the "natural" way of finding weights is to minimize *d *and we proved the following surprising

**Theorem 1**. *Let $\alpha_{k}=\left\lfloor \frac{\sqrt{1+8\left(k-1\right)}+1}{2}\right\rfloor .$The weight ω that minimizes d*(*S, T*) *has*

(2) $$\omega_{ij}=\left\{\begin{array}{cc} \frac{1}{k^{*}}, & \text{if}\;i<\alpha_{k^{*}},\;\;j\leq i,\\ & \text{or}\;i=a_{k^{*}},\;\;j\leq k^{*}-\frac{i\left(i-1\right)}{2}\\ 0, & \text{otherwise} \end{array}\right)$$

where k* is an integer and maximizes the function

(3) $$f\left(k\right)=\frac{1}{k}\left[\overset{\alpha_{k}-1}{\underset{i=1}{\sum }}\overset{i}{\underset{j=1}{\sum }}\left(n_{ij}+n_{ji}\right)+\overset{k-\frac{1}{2}\alpha_{k}\left(\alpha_{k}-1\right)}{\underset{j=1}{\sum }}\left({n_{\alpha }{}_{{}_{k}}}_{j}+{n_{j\alpha }}_{{}_{k}}\right)\right],$$

*where n_ij _is the number of gene pairs i-adjacent on S and j-adjacent on T*.

This suggests that uniform weights are appropriate for all (*i, j*) adjacencies up to a certain cutoff. Empirical work indicates that k* is of the order of $\sqrt{n},$where *n *is the number of vertices in the network and so the cutoff would be for *i *and *j *to be less than some value $\alpha \approx n^{\frac{1}{4}}.$ E.g., for a network with 100 vertices, it should suffice to consider 2- and 3-adjacencies, but 4-adjacencies need not be considered.

### The expected number of (*i, j*) adjacencies in two random networks

An essential step in studying gene clusters is to verify their significance. Random networks are often used to estimate the significance of clusters. In this section, we represent some characteristics of the expected number of (*i, j*) adjacencies in two random networks, which can then be used in evaluating cluster significance.

**Theorem 2**. *Let M be a randomly labelled square grid network with N vertices. Then the number of i adjacencies, n_i_, in the network M converges in distribution to the Poisson with parameter*

(4) $$E\left(n_{i}\right)=2i+O\left(\frac{1}{N}\right),$$

*the expected number of i adjacencies in the network M*.

*Proof*. Because *M *is a random square grid network, we can use a coordinate system to represent it. Vertices in the network correspond to the points in the plane with integer coordinates, x-coordinates being in the range 1,..., *m*, y-coordinates being in the range 1,..., *n*, where *N *= *mn*. Without loss of generality, we set *m *≤ *n*. Two vertices in the network are *i*-adjacent if the *L*_1 _distance between them in the integer coordinates is *i*.

Let $Y_{M}^{i}\left(u,v\right)$be 1 if vertices *u, v *are *i*-adjacent in the network *M *and 0 for otherwise. Then *n_i _*= $\sum_{\left(u,v\right)}Y_{M}^{i}\left(u,v\right).$Since most vertices have 4*i i*-adjacent neighbors, we can show that

(5) $$P\left(v,Y_{M}^{i}\left(u,v\right)=1|u\right)\;=\frac{4i}{mn-1}+O\left(\frac{1}{{\left(mn\right)}^{2}}\right),$$

where the error term is due to edge effects [9]. Since *N *= *mn*,

(6) $$\begin{array}{ccc} \; & P\left(Y_{M}^{i}\left(u,v\right)=1|\left(u,v\right)\right) & \\ = & P\left(v,Y_{M}^{i}\left(u,v\right)=1|u\right)P\left(u\right)\\ = & \frac{4i}{N\left(N-1\right)}+O\left(\frac{1}{N^{3}}\right) \end{array}$$

where the error term includes the edge effects detailed in equation(5). Then

(7) $$\begin{array}{ccc} E\left(n_{i}\right) & =\underset{\left(u,v\right)}{\sum }P\left(Y_{M}^{i}\left(u,v\right)=1|\left(u,v\right)\right) & \\ & =\underset{\left(u,v\right)}{\sum }\left[\frac{4i}{N\left(N-1\right)}+O\left(\frac{1}{N^{3}}\right)\right]\\ & =\frac{N\left(N-1\right)}{2}.\;\;\left[\frac{4i}{N\left(N-1\right)}+O\left(\frac{1}{N^{3}}\right)\right]\\ & =2i+O\left(\frac{1}{N}\right) \end{array}$$

Therefore, based on the proof of Theorem 2 in [10], we can conclude that *n_i _*converges in distribution to the Poisson with parameter *E*(*n_i_*) the expected number of *i *adjacencies in the network *M*.

**Theorem 3**. *For S and T two random square grid networks with the same N vertices, the number of pairs of vertices n_ij _that are i-adjacent in S and j-adjacent in T converges in distribution to the Poisson with parameter*

(8) $$E\left(n_{ij}\right)=8_{ij}+O\left(\frac{1}{N}\right),$$

*the expected number of *(*i, j*) *adjacencies in networks S and T*.

*Proof*. Let $Y_{S}^{i}\left(g,h\right)$be 1 if vertices *g, h *are *i*-adjacent in the random square grid network *S *and 0 otherwise. Similarly, define$Y_{T}^{j}\left(g,h\right)$to be 1 if vertices *g, h *are *j*-adjacent in the random square grid network *T *and 0 otherwise. Let $Y_{\left(S,T\right)}^{\left(i,j\right)}\left(g,h\right)$be 1 if vertices *g, h *are *i*-adjacent in *S *and *j*-adjacent in *T*. Otherwise $Y_{\left(S,T\right)}^{\left(i,j\right)}\left(g,h\right)=0.$ Because of the independence of *g, h *being *i*-adjacent in *S *and *j*-adjacent in *T*, the probability that *g *and *h *are (*i, j*)-adjacent in *S *and *T *is

(9) $$\begin{array}{c} P\left(Y_{\left(S,T\right)}^{\left(i,j\right)}\left(g,h\right)=1|\left(g,h\right)\right)\\ =P(Y_{S}^{i}\left(g,h\right)=1|\left(g,h\right)\;.\;P\left(Y_{T}^{j}\left(g,h\right)=1|\left(g,h\right)\right)\\ =\frac{16ij}{N^{2}{\left(N-1\right)}^{2}}+O\left(\frac{1}{N^{5}}\right) \end{array}$$

So the expected number of (*i, j*)-adjacencies in the two networks *S *and *T *is

(10) $$\begin{array}{ccc} E\left(n_{ij}\right) & =\underset{\left(g,h\right)\text{in}\;S,T}{\sum }P\left(Y_{\left(S,T\right)}^{\left(i,j\right)}\left(g,h\right)=1|\left(g,h\right)\right) & \\ & =\left[\frac{16ij}{N^{2}{\left(N-1\right)}^{2}}+O\left(\frac{1}{N^{5}}\right)\right].\underset{\left(g,h\right)\;\text{in}\;\;S,T}{\sum }1 \end{array}$$

The term $\sum_{\left(g,h\right)\;\text{in}\;S,T}1$in equation (10) represents the total number of (*g, h*) combinations in two networks *S *and *T *based on pairs of location of (*g, h*) in *S *and *T*. There are $\frac{1}{2}N\left(N-1\right)$ pairs of location possible for (*g, h*) in each of two networks and 2 alternatives for each gene pair (*g, h*) in *S *and *T*. So $\sum_{\left(g,h\right)\;\text{in}\;S,T}1=\frac{1}{2}N^{2}{\left(N-1\right)}^{2}.$ Hence, the expected number of (*i, j*)-adjacencies in the two networks *S *and *T *is

(11) $$\begin{array}{ccc} E\left(n_{ij}\right) & =\left[\frac{16ij}{N^{2}{\left(N-1\right)}^{2}}+O\left(\frac{1}{N^{5}}\right)\right]\;\;.\;\;\frac{N^{2}{\left(N-1\right)}^{2}}{2} & \\ & =8ij+O\left(\frac{1}{N}\right) \end{array}$$

Therefore, based on the proof of Theorem 2 in [10], we can conclude that *n_ij _*converges in distribution to the Poisson with parameter *E*(*n_ij_*), the expected number of (*i, j*) adjacencies in networks *S *and *T*.

More generally we can use the same techniques to prove Theorems 4 and 5:

**Theorem 4**. *Let D be the degree of a gene in the random genetic grid network, i.e. the number of *1*-adjacent neighbors of this gene in the network. For two random genetic lattice networks S and T with same genes, the number of pairs of genes n_ij _that are i-adjacent in S and j-adjacent in T converges in distribution to the Poisson with parameter*

(12) $$E\left(n_{ij}\right)=\frac{D^{2}ij}{2}+O\left(\frac{1}{N}\right)$$

Even for networks as small as 400, simulations indicate that the distribution of *n_ij _*is close to the Poisson in Theorem 4, for square (*D *= 4), hexagonal (*D *= 3), triangular (*D *= 6) grids as well as linear networks (*D *= 2). Looking beyond regular networks:

**Theorem 5**. *Let D_k_*(*M*) *be the number of k-adjacent gene pairs in the random network M. For two random networks S and T with same vertices, the number of pairs of vertices n_ij _that are i-adjacent in S and j-adjacent in T converges in distribution to the Poisson with parameter*

(13) $$E\left(n_{ij}\right)=\frac{D_{i}\left(S\right)D_{j}\left(T\right)}{2}+O\left(\frac{1}{N}\right)$$

## Results: genetic networks in *S. cerevisiae *and *S. pombe*

**Figure 5:**
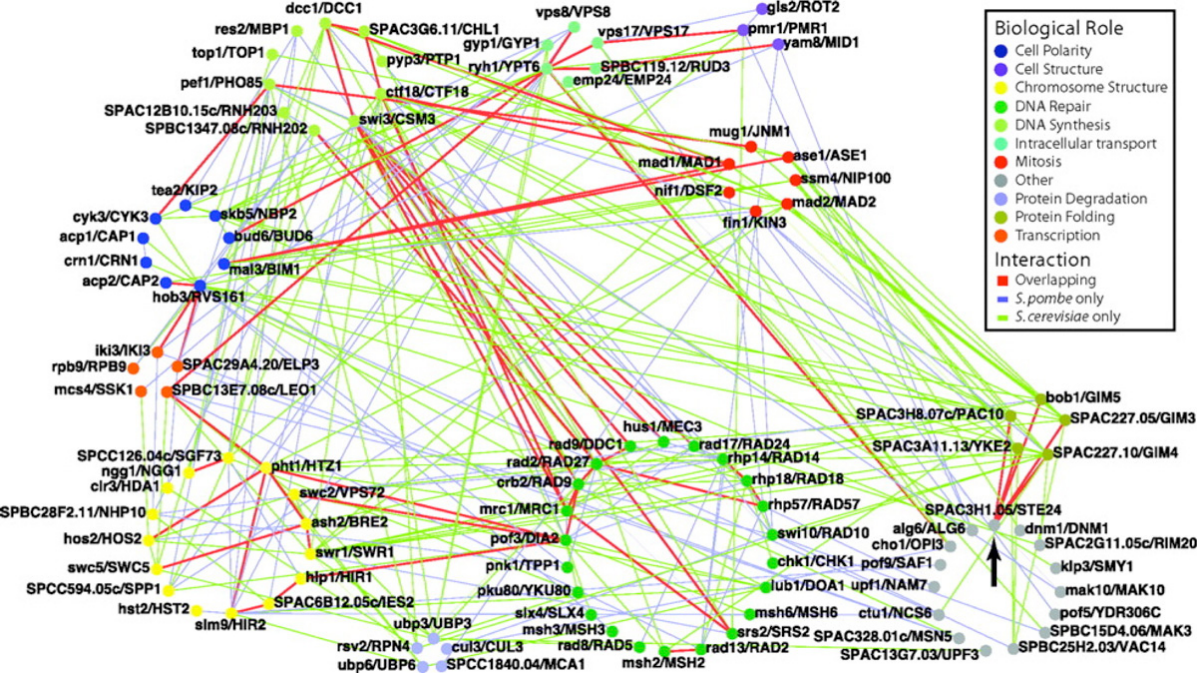
**Overlapping *S. cerevisiae *and *S. pombe *genetic networks**. Green edges: *S. cerevisiae *interactions only, blue edges: *S. pombe *only. Red edges: common to both networks. From [8]. ^©^2008 PNAS, S. Dixon *et al*.

**Figure 6:**
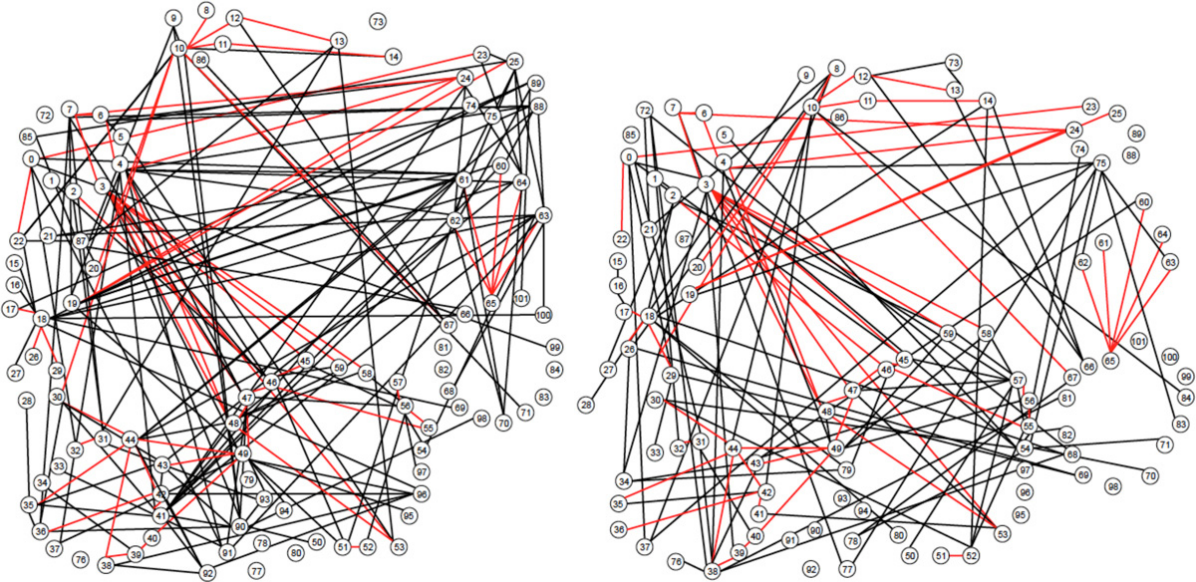
**Separate networks**. *S. cerevisiae *(left) and *S. pombe *(right) genetic networks. Red edges are common to both.

Dixon *et al. *[8] presented an extraordinary comparison of the genetic networks of *Saccharomyces cerevisiae *and *Schizosaccharomyces pombe*, two rather distant yeast genomes. Their results are summarized in their Figure 2, which we reproduce here as Figure 5. We separated the two overlapping networks based on the colours in this diagram, as depicted in Figure 6.

**Figure 7:**
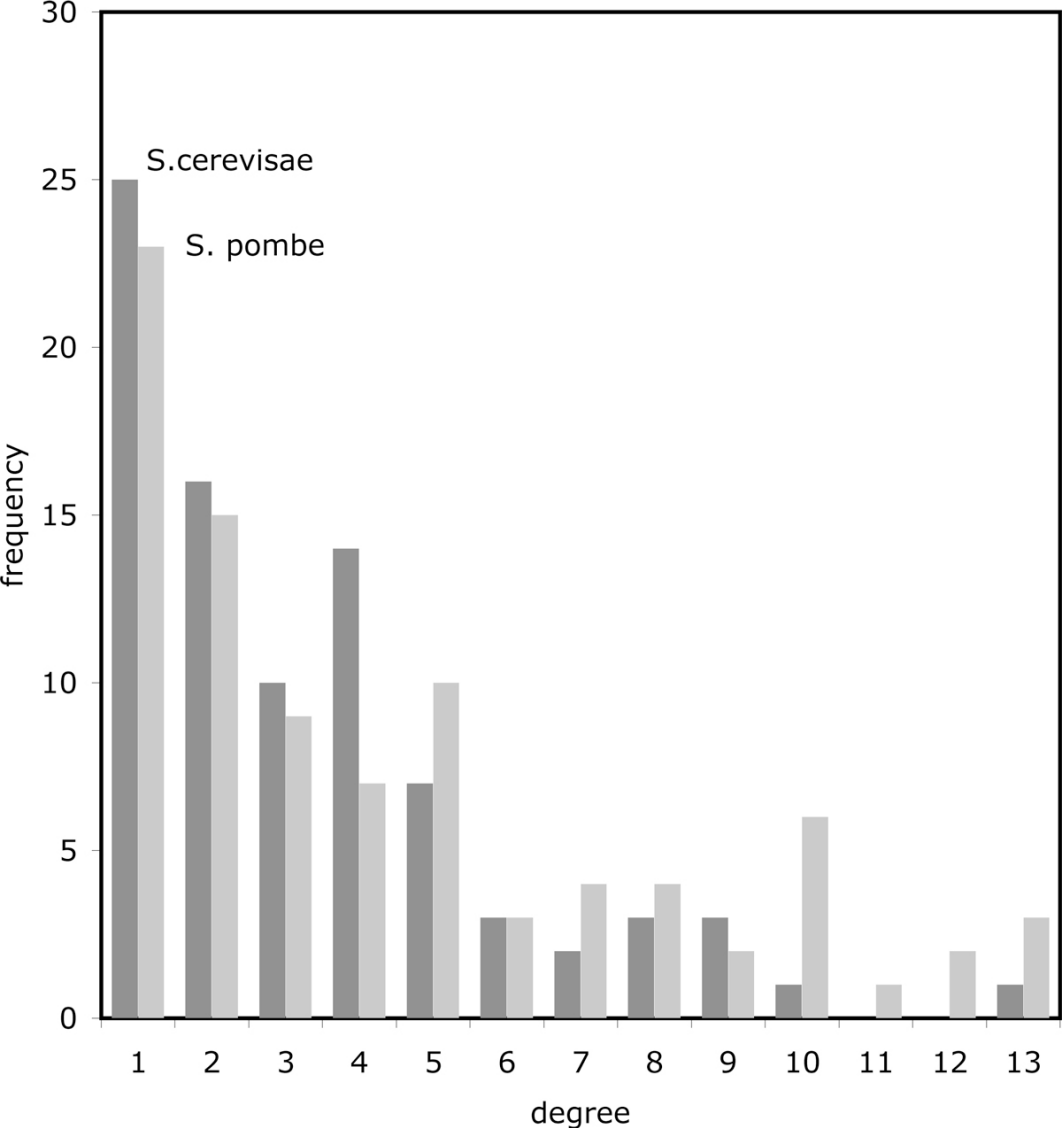
**Network characteristics**. Distribution of vertex degrees in yeast networks.

We compiled the graph-theoretical characteristics of these networks: number of vertices, average vertex degree, number of edges, and present them in Table 1. The details of the vertex degree distributions are given in Figure 7.

**Table 1:** Characteristics of comparative graph

yeast	vertices	average degree	edges
*S. cerevisiae*	89	4.36	194
*S. pombe*	85	3.34	142

in common	72		54

Number of vertices, their average degree and number of edges pertinent to each of the two yeasts.

**Figure 8:**
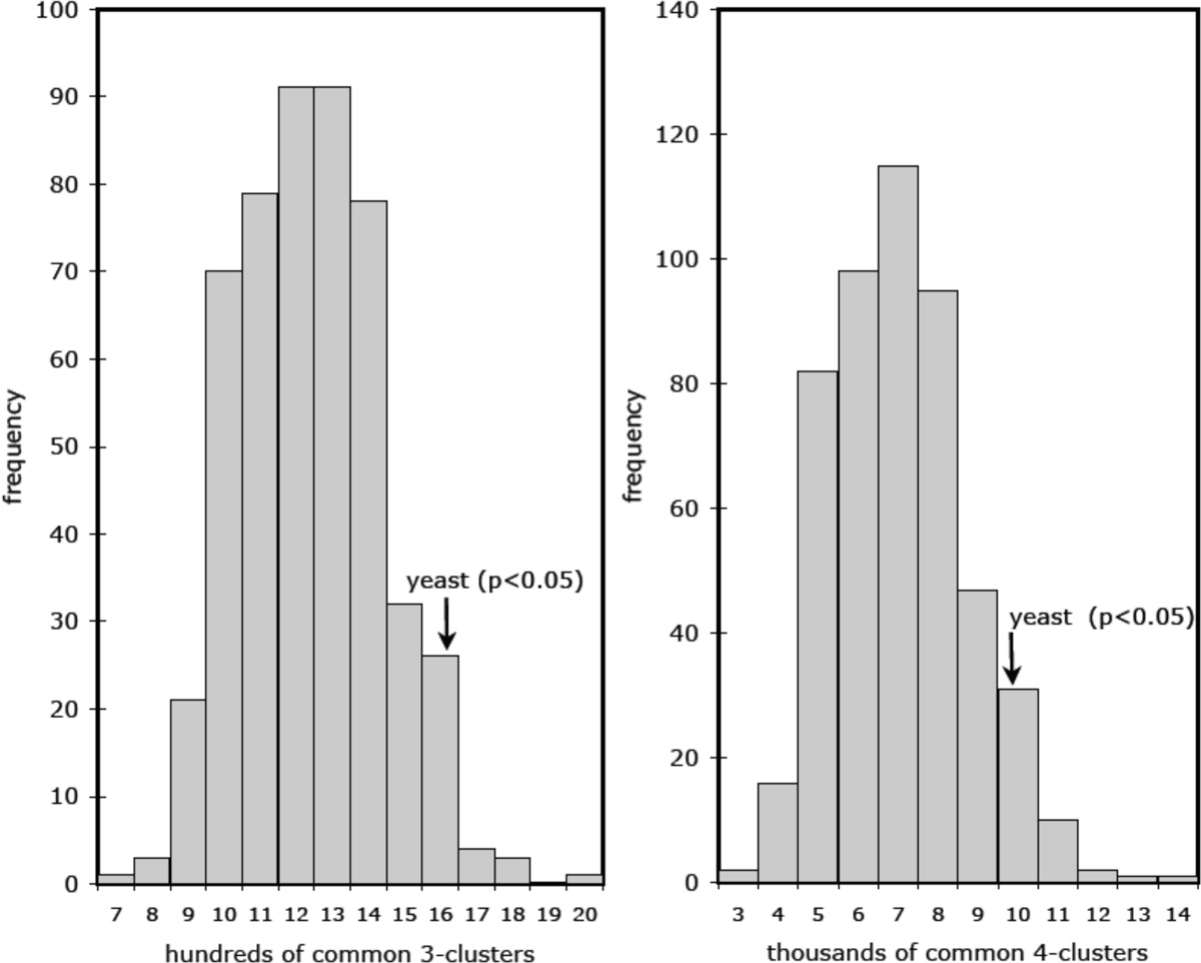
**Test of cluster frequencies**. Number of clusters containing three and four 2-adjacent genes in common, in 500 pairs of simulated networks with the same common 1-adjacencies as the two yeast networks.

We then carried out a number of simulations. First, we simulated random networks having the same statistical characteristics as in Table 1 and Figure 7. This showed the random networks to be deficient in (2,2)-, (3,3)- and (4,4)-clusters of genes compared to the yeast networks, under all of the (1,1)-, (2,2)- or (3,3)-adjacency criteria (see Table 2). In passing, we mention that the analysis of regular grid networks 7 earlier in this paper predicts very much smaller numbers of clusters than the random networks. Second, we fixed the common edges in both yeasts to initialize the random networks, and then generated the rest of the edges in conformity with Table 1 and Figure 7. This assured the (1,1)-adjacency results would be the same or close to the yeast results (see Table 2), but again the yeast networks showed a significant excess of clusters under (2,2)-adjacency. (The significance can be verified in Figure 8.)

**Table 2:** Characteristics of comparative graph

cluster size	graph	adjacency parameter*		
		**1-adj**.	**2-adj**.	**3-adj**.
2 genes	yeast	54	253	746
	random	4.5	99	581
	fixed common edges	53	217	767

3 genes	yeast	104	1,668	12,321
	random	1.2	442	8,909
	fixed common edges	101	1,292	12,185

4 genes	yeast	136	10,417	159,167
	random	0.3	1,741	104,339
	fixed common edges	132	7,567	160,821

Simulations showing heightened numbers of common 2-gene, 3-gene clusters 4-gene clusters under (2,2)-adjacency in the yeast network compared to random graphs.

**Figure 9:**
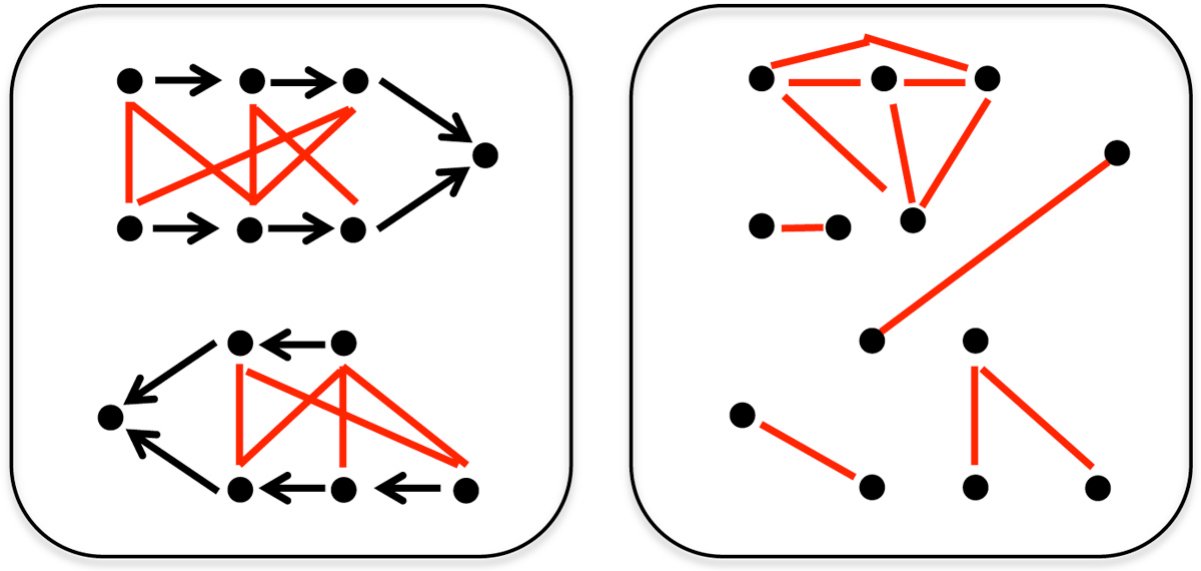
**Effect of synthetic lethality on cluster characteristics**. Comparison of a network intersection containing a number of parallel buffering pathways with a network without such pathways but with the same number of vertices and edges.

One of the factors responsible for the increase in clusters under 2-adjacency is the incidence of parallel buffering pathways in the genetic organization of these yeasts. Figure 9 illustrates how such pathways determine subgraphs in the network that are essentially bipartite. There are no 1-adjacencies among the genes in a single pathway, but the back-and-forth pattern of edges between the two sides of the bipartite structure ensures that under 2-adjacency, the genes in both pathways participate in clusters of various sizes.

As for the observation that 3-adjacency does not increase the number of clusters over random networks more than is achieved by fixing the common edges, this is partly explained by the fact that the yeast show only about 50% more clusters of each size than the random network, compared to the 250% -600% under 2-adjacency. Increasing the adjacency parameter in these networks simply results in large numbers of random clusters that swamp any subtle distinction between the fixed edge simulation and the yeast network.

## Conclusions

Generalized adjacency is a flexible but rigorous concept in the search for patterns of similarity among genetic networks. Although we analytically calculate properties of regular grid networks, e.g., linear, triangular, square and hexagonal grids, and though the average vertex degree of the empirically derived networks is in the same range as the hexagonal and square grids, the predicted number of clusters is much higher in the real data. This can be attributed in large part to the *dispersion *of the degree distribution, which is non-existent for the grids.

Of greater interest is the inability of random networks with the same characteristics as the real network to generate the same number of clusters. This is largely due to the small number of common adjacencies in the random networks, but even when this is forced to be the same, the yeast data showed an unexpected pattern of increased clustering under (2, 2)-adjacency, for all sizes of cluster (see Table 2). This was partly explicable in the way the networks were constructed using the synthetic lethals screen.

In conclusion, generalized adjacency is potentially a useful tool in exploring the special combinatorial structure of genetic networks.

## Competing interests

The authors declare that they have no competing interests.

## Authors' contributions

ZY and DS formulated the problem, carried out the calculations and simulations, and wrote the paper. Both authors read and approved the final manuscript.
